# Crystal structure of (*Z*)-3-allyl-5-(4-chloro­benzyl­idene)-2-sulfanyl­idene-1,3-thia­zolidin-4-one

**DOI:** 10.1107/S2056989015022689

**Published:** 2015-12-06

**Authors:** Rahhal El Ajlaoui, El Mostapha Rakib, Souad Mojahidi, Mohamed Saadi, Lahcen El Ammari

**Affiliations:** a Laboratoire de Chimie Organique et Analytique, Université Sultan Moulay Slimane, Faculté des Sciences et Techniques, Béni-Mellal, BP 523, Morocco; b Laboratoire de Chimie du Solide Appliquée, Faculté des Sciences, Université Mohammed V de Rabat, Avenue Ibn Battouta, BP 1014, Rabat, Morocco

**Keywords:** crystal structure, rhodanine-based mol­ecules, pharmacological activity, biological activity, 1,3-thia­zolidin-4-one

## Abstract

In the title compound, C_13_H_10_ClNOS_2_, the dihedral angle between the rhodanine (r.m.s. deviation = 0.008 Å) and 4-chloro­benzyl­idene rings is 1.79 (11)°. The allyl group attached to the N atom, which lies almost perpendicular to the rhodanine ring, is disordered over two orientations in a 0.519 (13):0.481 (13) ratio. A short intra­molecular C—H⋯S inter­action closes an *S*(6) ring. In the crystal, mol­ecules are linked by π–π stacking inter­actions [centroid–centroid separation = 3.600 (15) Å], generating inversion dimers.

## Related literature   

For a related structure and background to the pharmacological and biological activities of rhodanine-based mol­ecules, see: El Ajlaoui *et al.* (2015 ▸).
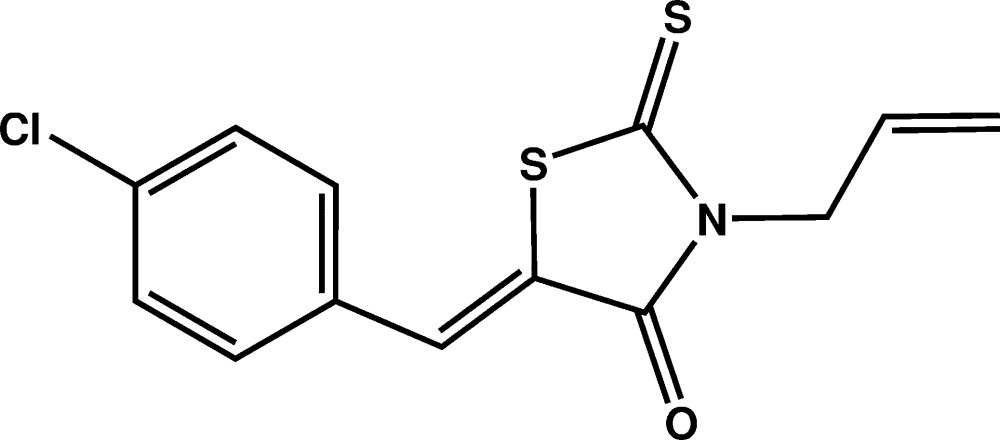


## Experimental   

### Crystal data  

C_13_H_10_ClNOS_2_
*M* *_r_* = 295.79
Triclinic, 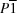

*a* = 7.6197 (8) Å
*b* = 7.9849 (7) Å
*c* = 13.0624 (14) Å
α = 77.600 (5)°
β = 77.996 (5)°
γ = 61.954 (4)°
*V* = 679.76 (12) Å^3^
*Z* = 2
Mo *K*α radiation
μ = 0.57 mm^−1^
*T* = 296 K
0.37 × 0.25 × 0.21 mm

### Data collection  

Bruker X8 APEX CCD diffractometer
Absorption correction: multi-scan (*SADABS*; Bruker, 2009 ▸) *T*_min_ = 0.656, *T*_max_ = 0.746
24189 measured reflections
3249 independent reflections
2199 reflections with *I* > 2σ(*I*)
*R*_int_ = 0.038

### Refinement  

*R*[*F* ^2^ > 2σ(*F* ^2^)] = 0.047
*wR*(*F* ^2^) = 0.144
*S* = 1.04
3249 reflections
182 parameters
3 restraints
H-atom parameters constrained
Δρ_max_ = 0.38 e Å^−3^
Δρ_min_ = −0.35 e Å^−3^

### 

Data collection: *APEX2* (Bruker, 2009 ▸); cell refinement: *SAINT* (Bruker, 2009 ▸); data reduction: *SAINT*; program(s) used to solve structure: *SHELXS97* (Sheldrick, 2008 ▸); program(s) used to refine structure: *SHELXL2014* (Sheldrick, 2015 ▸); molecular graphics: *ORTEPIII* (Burnett & Johnson, 1996 ▸) and *ORTEP-3 for Windows* (Farrugia, 2012 ▸); software used to prepare material for publication: *publCIF* (Westrip, 2010 ▸).

## Supplementary Material

Crystal structure: contains datablock(s) I. DOI: 10.1107/S2056989015022689/hb7551sup1.cif

Structure factors: contains datablock(s) I. DOI: 10.1107/S2056989015022689/hb7551Isup2.hkl

Supporting information file. DOI: 10.1107/S2056989015022689/hb7551Isup3.cml

**Fig. 1.**
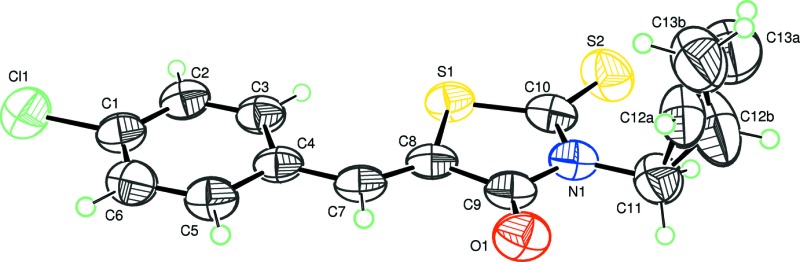
Plot of the molecule of the title compound with displacement ellipsoids drawn at the 50% probability level. H atoms are represented as small circles.

. DOI: 10.1107/S2056989015022689/hb7551fig1.tif
Plot of the mol­ecule of the title compound with displacement ellipsoids drawn at the 50% probability level. H atoms are represented as small circles.

**Fig. 2.**
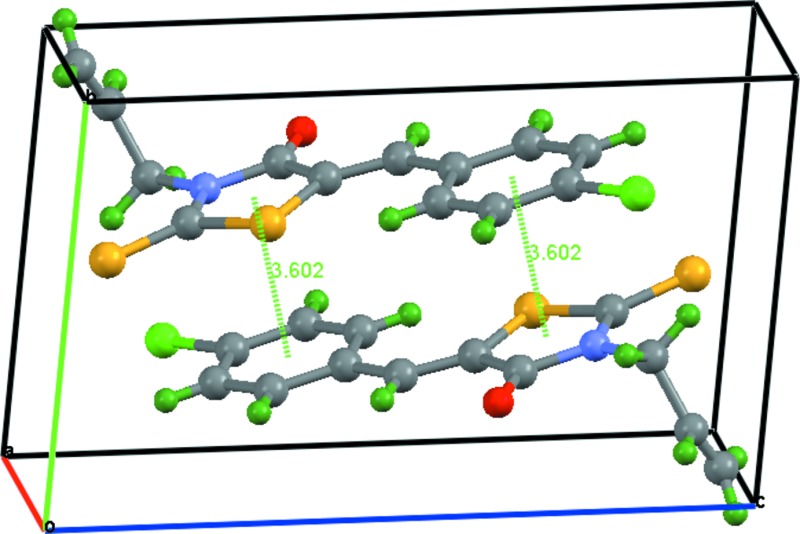
Crystal packing for the title compound showing hydrogen bonds as dashed lines between inversion-related molecules.

. DOI: 10.1107/S2056989015022689/hb7551fig2.tif
Crystal packing for the title compound showing hydrogen bonds as dashed lines between inversion-related mol­ecules.

CCDC reference: 1439050

Additional supporting information:  crystallographic information; 3D view; checkCIF report

## Figures and Tables

**Table 1:** Hydrogen-bond geometry (Å, °)

*D*—H⋯*A*	*D*—H	H⋯*A*	*D*⋯*A*	*D*—H⋯*A*
C3—H3⋯S1	0.93	2.55	3.254 (3)	133

## supplementary crystallographic information

### S1. Comment

As part of our ongoing studies of rhodanine derivatives, we now describe the title compound.

The molecule of the title compound is build up from a rhodanine ring (S1–N1–C8–C9–C10) linked to an disordered allyl group (48%/52%) (C11–C12AC12B–C13AC13B) and at the nitrogen atom and to a 4-chlorobenzylidene ring system (C1 to C6) as shown in Fig.1. The mean plane through the rhodanine ring is almost perpendicular to the allyl group and makes a dihedral angle of 1.79 (11)° with the 4-chlorobenzylidene ring system. Nearly the same structure is observed by El Ajlaoui *et al.* 2015 in (*Z*)-3-Allyl-5-(4-methyl-benzylidene)-2- thioxothiazolidin-4-one.

The cohesion of the crystal structure is ensured by π—π interaction between molecules forming inversion dimers as shown in Fig.2.

### S2. Experimental

To a solution of 3-allylrhodanine (1.15 mmol, 0.2 g) in 10 ml of THF, (4-chlorobenzylidene)-4-methyl-5-oxopyrazolidin-2-ium-1-ide (1.38 mmol) was added. The mixture was refluxed for 8 h, monitored by TLC, the reaction completed and a yellow spot (TLC Rf = 0.3, using hexane/ethyl acetate 1:9) was generated cleanly. The solvent was evaporated *in vacuo*. The crude product was purified on silica gel using hexane: ethyl acetate (1/9) as eluent. The title compound was recrystallized from ethanol (Yield: 72%, m.p.: 371 K).

### S3. Refinement

H atoms were located in a difference map and treated as riding with C–H = 0.97 Å and C–H = 0.93 Å for methylene and aromatic, respectively. All hydrogen with *U*_iso_(H) = 1.2 *U*_eq_ for methylene and aromatic. The reflection (0 0 1) affected by the beam-stop is removed during refinement.

### Crystal data

**Table:**

C_13_H_10_ClNOS_2_	*F*(000) = 304
*M**_r_* = 295.79	*D*_x_ = 1.445 Mg m^−^^3^
Triclinic, *P*1	Melting point: 371 K
*a* = 7.6197 (8) Å	Mo *K*α radiation, λ = 0.71073 Å
*b* = 7.9849 (7) Å	Cell parameters from 3249 reflections
*c* = 13.0624 (14) Å	θ = 2.9–27.9°
α = 77.600 (5)°	µ = 0.57 mm^−^^1^
β = 77.996 (5)°	*T* = 296 K
γ = 61.954 (4)°	Block, colourless
*V* = 679.76 (12) Å^3^	0.37 × 0.25 × 0.21 mm
*Z* = 2	

### Data collection

**Table:**

Bruker X8 APEX CCD diffractometer	3249 independent reflections
Radiation source: fine-focus sealed tube	2199 reflections with *I* > 2σ(*I*)
Graphite monochromator	*R*_int_ = 0.038
φ and ω scans	θ_max_ = 27.9°, θ_min_ = 2.9°
Absorption correction: multi-scan (*SADABS*; Bruker, 2009)	*h* = −10→9
*T*_min_ = 0.656, *T*_max_ = 0.746	*k* = −10→10
24189 measured reflections	*l* = −17→17

### Refinement

**Table:**

Refinement on *F*^2^	3 restraints
Least-squares matrix: full	Hydrogen site location: inferred from neighbouring sites
*R*[*F*^2^ > 2σ(*F*^2^)] = 0.047	H-atom parameters constrained
*wR*(*F*^2^) = 0.144	*w* = 1/[σ^2^(*F*_o_^2^) + (0.0576*P*)^2^ + 0.2968*P*] where *P* = (*F*_o_^2^ + 2*F*_c_^2^)/3
*S* = 1.04	(Δ/σ)_max_ = 0.001
3249 reflections	Δρ_max_ = 0.38 e Å^−^^3^
182 parameters	Δρ_min_ = −0.35 e Å^−^^3^

### Special details

**Table:**

Geometry. All e.s.d.'s (except the e.s.d. in the dihedral angle between two l.s. planes) are estimated using the full covariance matrix. The cell e.s.d.'s are taken into account individually in the estimation of e.s.d.'s in distances, angles and torsion angles; correlations between e.s.d.'s in cell parameters are only used when they are defined by crystal symmetry. An approximate (isotropic) treatment of cell e.s.d.'s is used for estimating e.s.d.'s involving l.s. planes.

### Fractional atomic coordinates and isotropic or equivalent isotropic displacement parameters (Å^2^)

**Table:**

	*x*	*y*	*z*	*U*_iso_*/*U*_eq_	Occ. (<1)
C1	−0.1200 (4)	0.7895 (3)	0.6827 (2)	0.0698 (7)	
C2	−0.0835 (4)	0.7296 (4)	0.5860 (2)	0.0709 (7)	
H2	−0.1709	0.6939	0.5668	0.085*	
C3	0.0823 (4)	0.7228 (3)	0.5181 (2)	0.0652 (6)	
H3	0.1060	0.6822	0.4528	0.078*	
C4	0.2174 (3)	0.7752 (3)	0.54439 (19)	0.0574 (6)	
C5	0.1746 (4)	0.8355 (3)	0.6429 (2)	0.0678 (7)	
H5	0.2612	0.8714	0.6627	0.081*	
C6	0.0086 (5)	0.8438 (4)	0.7117 (2)	0.0762 (7)	
H6	−0.0170	0.8853	0.7769	0.091*	
C7	0.3946 (4)	0.7721 (3)	0.4765 (2)	0.0592 (6)	
H4	0.4665	0.8149	0.5045	0.071*	
C8	0.4733 (4)	0.7186 (3)	0.38062 (19)	0.0584 (6)	
C9	0.6604 (4)	0.7264 (3)	0.3269 (2)	0.0634 (6)	
C10	0.5892 (4)	0.6044 (4)	0.2013 (2)	0.0712 (7)	
C11	0.8918 (5)	0.6605 (5)	0.1606 (3)	0.0912 (9)	
H11A	0.9341	0.5757	0.1079	0.109*	
H11B	1.0013	0.6194	0.2015	0.109*	
C12A	0.8259 (17)	0.8701 (16)	0.1084 (6)	0.100 (3)	0.519 (13)
H12A	0.8190	0.9597	0.1463	0.120*	0.519 (13)
C13A	0.779 (2)	0.925 (2)	0.0099 (6)	0.131 (4)	0.519 (13)
H13A	0.7852	0.8369	−0.0287	0.157*	0.519 (13)
H13B	0.7393	1.0524	−0.0202	0.157*	0.519 (13)
C12B	0.8935 (18)	0.8039 (12)	0.0686 (7)	0.144 (6)	0.481 (13)
H12B	1.0008	0.7686	0.0151	0.172*	0.481 (13)
C13B	0.747 (2)	0.9822 (14)	0.0591 (12)	0.140 (6)	0.481 (13)
H13C	0.6384	1.0200	0.1117	0.168*	0.481 (13)
H13D	0.7530	1.0688	−0.0002	0.168*	0.481 (13)
N1	0.7137 (3)	0.6620 (3)	0.22888 (17)	0.0667 (5)	
O1	0.7595 (3)	0.7794 (3)	0.36081 (16)	0.0818 (6)	
S1	0.38675 (10)	0.63202 (10)	0.29984 (6)	0.0703 (2)	
S2	0.61487 (17)	0.51989 (15)	0.09357 (7)	0.1047 (3)	
Cl1	−0.33030 (13)	0.79962 (14)	0.76777 (7)	0.1017 (3)	

### Atomic displacement parameters (Å^2^)

**Table:**

	*U*^11^	*U*^22^	*U*^33^	*U*^12^	*U*^13^	*U*^23^
C1	0.0656 (15)	0.0542 (13)	0.0814 (17)	−0.0180 (11)	−0.0213 (13)	−0.0017 (12)
C2	0.0635 (15)	0.0670 (15)	0.0856 (18)	−0.0260 (12)	−0.0248 (14)	−0.0091 (13)
C3	0.0675 (15)	0.0583 (13)	0.0731 (15)	−0.0225 (12)	−0.0262 (12)	−0.0119 (11)
C4	0.0618 (13)	0.0388 (10)	0.0715 (14)	−0.0163 (9)	−0.0270 (11)	−0.0041 (10)
C5	0.0759 (17)	0.0565 (13)	0.0795 (17)	−0.0280 (12)	−0.0257 (14)	−0.0138 (12)
C6	0.0841 (19)	0.0632 (15)	0.0758 (17)	−0.0217 (14)	−0.0203 (15)	−0.0153 (13)
C7	0.0652 (14)	0.0444 (11)	0.0755 (15)	−0.0228 (10)	−0.0310 (12)	−0.0052 (10)
C8	0.0647 (14)	0.0441 (11)	0.0727 (15)	−0.0224 (10)	−0.0320 (12)	−0.0019 (10)
C9	0.0704 (15)	0.0502 (12)	0.0743 (16)	−0.0265 (11)	−0.0300 (12)	0.0017 (11)
C10	0.0826 (17)	0.0618 (14)	0.0712 (16)	−0.0280 (13)	−0.0312 (14)	−0.0024 (12)
C11	0.094 (2)	0.101 (2)	0.082 (2)	−0.0513 (19)	−0.0114 (17)	−0.0002 (17)
C12A	0.118 (7)	0.113 (8)	0.074 (5)	−0.068 (7)	0.019 (5)	−0.014 (5)
C13A	0.134 (10)	0.109 (9)	0.136 (9)	−0.044 (8)	−0.018 (8)	−0.011 (7)
C12B	0.221 (15)	0.113 (8)	0.077 (7)	−0.075 (9)	0.033 (8)	−0.022 (6)
C13B	0.147 (9)	0.103 (8)	0.092 (9)	−0.008 (7)	0.022 (7)	−0.009 (6)
N1	0.0715 (13)	0.0601 (11)	0.0714 (13)	−0.0293 (10)	−0.0238 (11)	−0.0003 (10)
O1	0.0901 (13)	0.0898 (13)	0.0922 (13)	−0.0563 (11)	−0.0284 (11)	−0.0093 (10)
S1	0.0730 (4)	0.0733 (4)	0.0787 (4)	−0.0340 (3)	−0.0272 (3)	−0.0173 (3)
S2	0.1285 (8)	0.1284 (8)	0.0783 (5)	−0.0645 (6)	−0.0206 (5)	−0.0303 (5)
Cl1	0.0793 (5)	0.1100 (7)	0.1008 (6)	−0.0346 (5)	−0.0040 (4)	−0.0102 (5)

### Geometric parameters (Å, º)

**Table:**

C1—C2	1.374 (4)	C9—N1	1.392 (3)
C1—C6	1.384 (4)	C10—N1	1.366 (3)
C1—Cl1	1.728 (3)	C10—S2	1.626 (3)
C2—C3	1.370 (4)	C10—S1	1.749 (3)
C2—H2	0.9300	C11—N1	1.457 (4)
C3—C4	1.402 (3)	C11—C12B	1.4743 (10)
C3—H3	0.9300	C11—C12A	1.543 (11)
C4—C5	1.392 (3)	C11—H11A	0.9700
C4—C7	1.445 (4)	C11—H11B	0.9700
C5—C6	1.373 (4)	C12A—C13A	1.3334 (10)
C5—H5	0.9300	C12A—H12A	0.9300
C6—H6	0.9300	C13A—H13A	0.9300
C7—C8	1.338 (3)	C13A—H13B	0.9300
C7—H4	0.9300	C12B—C13B	1.3333 (10)
C8—C9	1.475 (4)	C12B—H12B	0.9300
C8—S1	1.749 (2)	C13B—H13C	0.9300
C9—O1	1.211 (3)	C13B—H13D	0.9300
			
C2—C1—C6	120.6 (3)	N1—C10—S2	127.3 (2)
C2—C1—Cl1	119.4 (2)	N1—C10—S1	110.9 (2)
C6—C1—Cl1	119.9 (2)	S2—C10—S1	121.89 (17)
C3—C2—C1	119.5 (2)	N1—C11—C12B	125.5 (5)
C3—C2—H2	120.2	N1—C11—C12A	104.4 (5)
C1—C2—H2	120.2	N1—C11—H11A	110.9
C2—C3—C4	121.8 (2)	C12A—C11—H11A	110.9
C2—C3—H3	119.1	N1—C11—H11B	110.9
C4—C3—H3	119.1	C12A—C11—H11B	110.9
C5—C4—C3	116.8 (2)	H11A—C11—H11B	108.9
C5—C4—C7	118.5 (2)	C13A—C12A—C11	121.3 (11)
C3—C4—C7	124.7 (2)	C13A—C12A—H12A	119.4
C6—C5—C4	122.1 (2)	C11—C12A—H12A	119.4
C6—C5—H5	119.0	C12A—C13A—H13A	120.0
C4—C5—H5	119.0	C12A—C13A—H13B	120.0
C5—C6—C1	119.1 (3)	H13A—C13A—H13B	120.0
C5—C6—H6	120.4	C13B—C12B—C11	122.4 (11)
C1—C6—H6	120.4	C13B—C12B—H12B	118.8
C8—C7—C4	131.4 (2)	C11—C12B—H12B	118.8
C8—C7—H4	114.3	C12B—C13B—H13C	120.0
C4—C7—H4	114.3	C12B—C13B—H13D	120.0
C7—C8—C9	121.4 (2)	H13C—C13B—H13D	120.0
C7—C8—S1	129.3 (2)	C10—N1—C9	116.3 (2)
C9—C8—S1	109.29 (18)	C10—N1—C11	123.1 (3)
O1—C9—N1	122.6 (3)	C9—N1—C11	120.6 (2)
O1—C9—C8	126.4 (3)	C8—S1—C10	92.62 (12)
N1—C9—C8	110.9 (2)		

### Hydrogen-bond geometry (Å, º)

**Table:**

*D*—H···*A*	*D*—H	H···*A*	*D*···*A*	*D*—H···*A*
C3—H3···S1	0.93	2.55	3.254 (3)	133
