# Supplementary figures and images for: Crystal structure of (Z)-3-allyl-5-(4-chloro­benzyl­idene)-2-sulfanyl­idene-1,3-thia­zolidin-4-one
Source: Acta Crystallogr E Crystallogr Commun. 2015 Dec 6;71(Pt 12):o1012. doi: 10.1107/S2056989015022689 (PMC4719951; doi:10.1107/S2056989015022689)

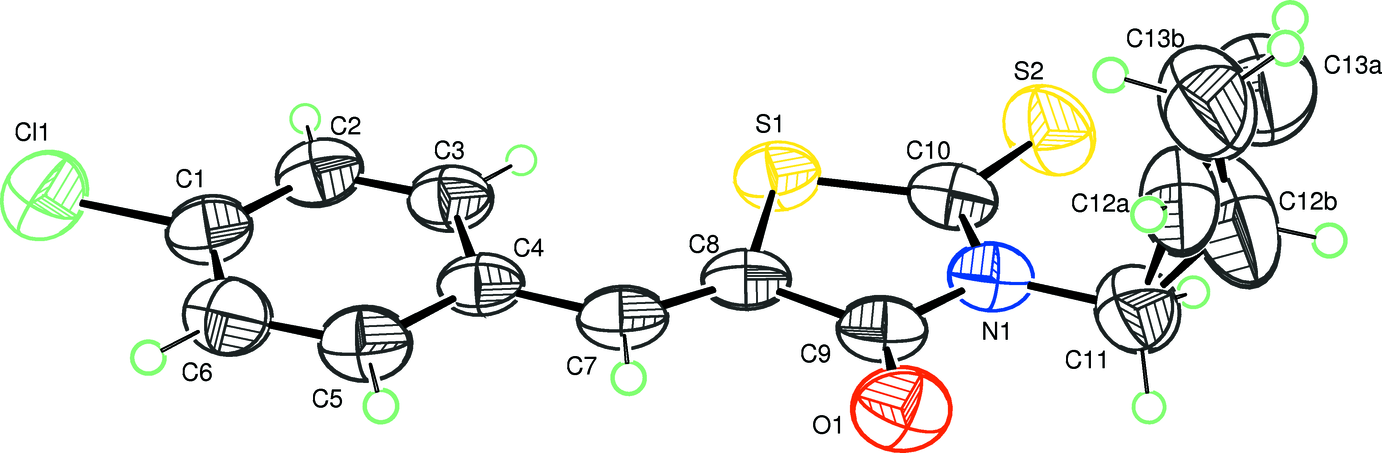

Supplement: Supplementary file 4 [file e-71-o1012-fig1.tif]

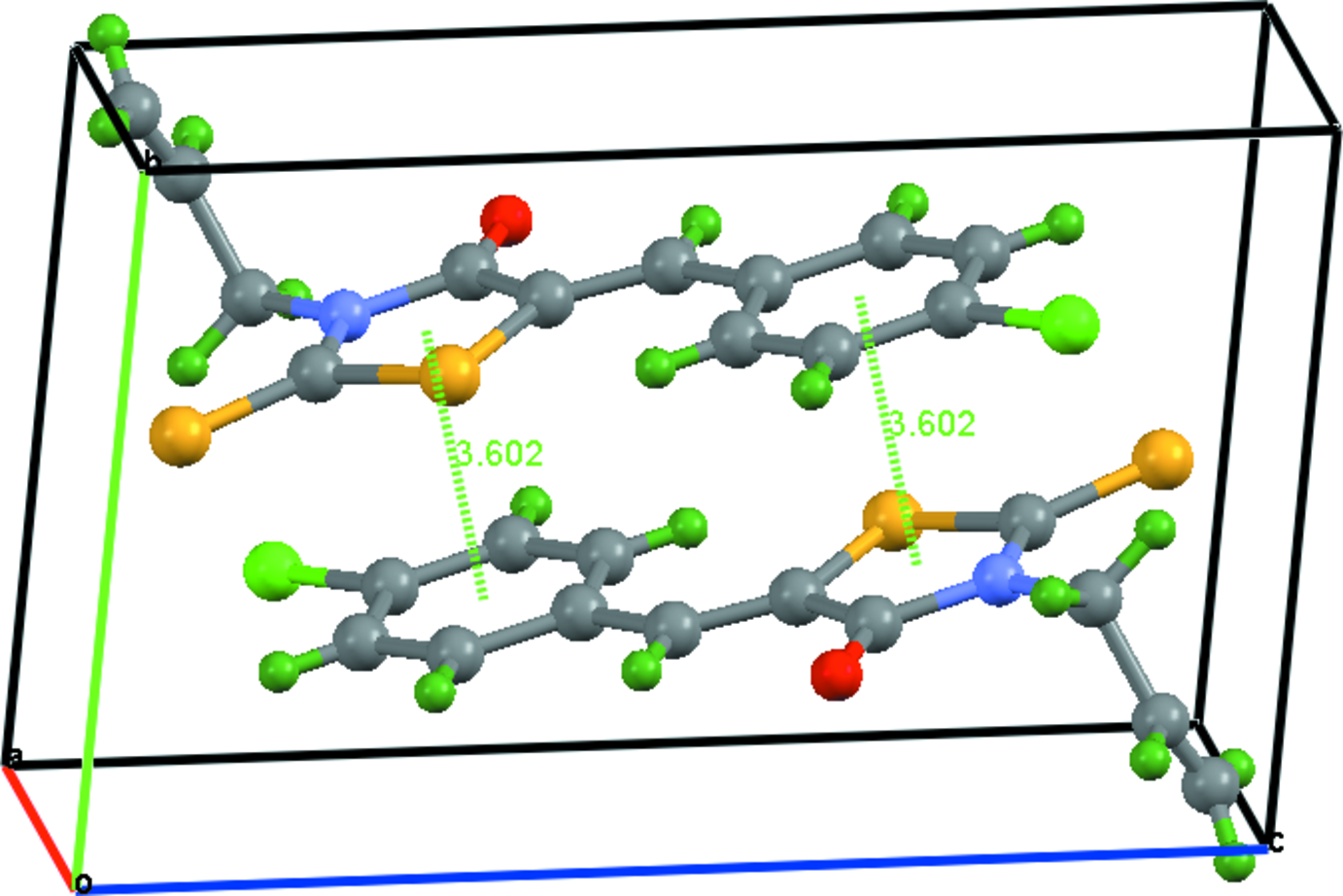

Supplement: Supplementary file 5 [file e-71-o1012-fig2.tif]
